# Characterization of the selection of medicines for the Brazilian primary health care

**DOI:** 10.11606/S1518-8787.2017051007065

**Published:** 2017-09-22

**Authors:** Margô Gomes de Oliveira Karnikowski, Dayani Galato, Micheline Marie Milward de Azevedo Meiners, Emília Vitória da Silva, Letícia Farias Gerlack, Ângelo José Gonçalves Bós, Silvana Nair Leite, Juliana Álvares, Ione Aquemi Guibu, Orlando Mario Soeiro, Karen Sarmento Costa, Ediná Alves Costa, Augusto Afonso Guerra, Francisco de Assis Acurcio

**Affiliations:** I Curso de Farmácia. Faculdade de Ceilândia. Universidade de Brasília. Brasília, DF, Brasil; II Programa de Pós-graduação em Ciências e Tecnologias em Saúde. Faculdade de Ceilândia. Universidade de Brasília. Brasília, DF, Brasil; III Pontifícia Universidade Católica do Rio Grande do Sul. Porto Alegre, RS, Brasil; IV Departamento de Ciências Farmacêuticas. Universidade Federal de Santa Catarina. Florianópolis, SC, Brasil; V Departamento de Farmácia Social. Faculdade de Farmácia. Universidade Federal de Minas Gerais. Belo Horizonte, MG, Brasil; VI Departamento de Saúde Coletiva. Faculdade de Ciências Médicas. Santa Casa de São Paulo. São Paulo, SP, Brasil; VII Faculdade de Ciências Farmacêuticas. Pontifícia Universidade Católica de Campinas. Campinas, SP, Brasil; VIII Núcleo de Estudos de Políticas Públicas. Universidade Estadual de Campinas. Campinas, SP, Brasil; IX Programa de Pós-Graduação em Saúde Coletiva. Departamento de Saúde Coletiva. Faculdade de Ciências Médicas. Universidade Estadual de Campinas. Campinas, SP, Brasil; X Programa de Pós-Graduação em Epidemiologia. Faculdade de Medicina. Universidade Federal do Rio Grande do Sul. Porto Alegre, RS, Brasil; XI Instituto de Saúde Coletiva. Universidade Federal da Bahia. Salvador, BA, Brasil

**Keywords:** Drugs, Essential, classification, Pharmacy and Therapeutics Committee, Pharmaceutical Services, National Policy of Pharmaceutical Assistance, Primary Health Care, Health Services Research, Unified Health System, Medicamentos Essenciais, classificação, Comitê de Farmácia e Terapêutica, Assistência Farmacêutica, Política Nacional de Assistência Farmacêutica, Atenção Primária à Saúde, Pesquisa sobre Serviços de Saúde, Sistema Único de Saúde

## Abstract

**OBJECTIVE:**

To characterize the process of selection of medicines for primary health care in the Brazilian regions.

**METHODS:**

This article is part of the *Pesquisa Nacional sobre Acesso, Utilização e Promoção do Uso Racional de Medicamentos – Serviços, 2015* (PNAUM – National Survey on Access, Use and Promotion of Rational Use of Medicines – Services, 2015), a cross-sectional study that consisted of an information gathering in a sample of cities in the five regions of Brazil. The data used were collected by interviews with those responsible for pharmaceutical services (PS) (n = 506), professionals responsible for the dispensing of medicines (n = 1,139), and physicians (n = 1,558). To evaluate the difference between ratios, we adopted the Chi-square test for complex samples. The differences between the averages were analyzed in generalized linear models with F-test with Bonferroni correction for multiple comparisons. The analyses considered significant had p≤0.05.

**RESULTS:**

The professionals responsible for pharmaceutical services reported non-existence of a formally constituted Pharmacy and Therapeutics Committee (PTC) (12.5%). They claimed to have an updated (80.4%) list of Essential Medicines (85.3%) and being active participants of this process (88.2%). However, in the perception of respondents, the list only partially (70.1%) meets the health demands. Of the interviewed professionals responsible for the dispensing of medicines, only 16.6% were pharmacists; even so, 47.8% reported to know the procedures to change the list. From the perspective of most of these professionals (70.9%), the list meets the health demands of the city. Among physicians, only 27.2% reported to know the procedures to change the list, but 76.5% would have some claim to change it. Most of them reported to base their claims in clinical experiences (80.0%). For 13.0% of them, the list meets the health demands.

**CONCLUSIONS:**

As this is the first national survey of characterization of the process of selection of medicines within primary health care, it brings unpublished data for the assessment of policies related to medicines in Brazil.

## RESUMO

**OBJETIVO:**

Caracterizar o processo de seleção de medicamentos na atenção primária à saúde, nas regiões brasileiras.

**MÉTODOS:**

Artigo integrante da Pesquisa Nacional sobre Acesso, Utilização e Promoção do Uso Racional de Medicamentos – Serviços 2015, um estudo transversal que envolveu o levantamento de informações numa amostra de municípios das cinco regiões do Brasil. Os dados utilizados foram coletados por meio de entrevistas com os responsáveis pela assistência farmacêutica (n = 506), profissionais que realizam a dispensação de medicamentos (n = 1.139) e médicos (n = 1.558). Para avaliar a diferença entre as proporções foi adotado o teste do qui-quadrado para amostras complexas. As diferenças entre as médias foram analisadas em modelos lineares generalizados, com teste F com correção de Bonferroni para comparações múltiplas. Foram significativas as análises com valores de p ≤ 0,05.

**RESULTADOS:**

Os responsáveis da assistência farmacêutica relataram não haver Comissão de Farmácia e Terapêutica formalmente constituída (12,5%). Eles afirmaram possuir uma Lista de Medicamentos Essenciais (85,3%) atualizada (80,4%), e serem participantes deste processo (88,2%). Contudo, na percepção dos entrevistados a Lista atende apenas parcialmente (70,1%) as demandas de saúde. Dos profissionais que realizam a dispensação de medicamentos entrevistados, apenas 16,6% eram farmacêuticos, mesmo assim, 47,8% referiram conhecer os procedimentos para mudança da lista. Na perspectiva da maior parte desses profissionais (70,9%), a lista atende as demandas de saúde do município. Dentre os médicos apenas 27,2% declararam ter conhecimento dos procedimentos para mudança da lista, mas 76,5% teriam alguma reivindicação de alteração. A maior parte reporta que baseia suas reivindicações em experiências clínicas (80,0%); para 13,0% deles, a lista atende às demandas de saúde.

**CONCLUSÕES:**

Por tratar-se da primeira pesquisa nacional de caracterização do processo de seleção de medicamentos no âmbito da atenção primária à saúde, traz dados inéditos para a avaliação das políticas relacionadas com medicamentos no Brasil.

## INTRODUCTION

Selection of medicines is a guiding axis for actions of planning and organization of pharmaceutical services (PS)^6^. If well conducted, it provides economic gains to the Brazilian Unified Health System (SUS), as well as to the access to effective, safe, and cost-effective medicines for the patients of the System^6^. However, in Brazil, weaknesses and barriers can be observed in the selection process, which are critical for the implementation of access and rational use of medicines by the population^3^.

For the selection to occur adequately, it must meet certain requirements, such as the establishment of a Pharmacy and Therapeutics Committee (PTC), which is responsible for the technical, political, and administrative conduction of the process^4^. The main product of this process is a list of essential medicines (LME), from which should derive the therapeutic form and clinical guidelines. The LME, based on an epidemiological perspective, must contain essential medicines for public health, having their effectiveness, safety, and quality assured and being prescribed and used rationally^11^.

The selection has the potential to produce results that guide both clinical conducts and management activities related to medicines provided by SUS^11^. To this end, the selection process must involve the articulation of different actors, especially health managers and professionals, both nationally and locally^3^.

According to the *Política Nacional de Medicamentos* (PNM – National Drug Policy)^7^, the existence of a *Relação Nacional de Medicamentos Essenciais* (Rename – National List of Essential Medicines) does not exempt the responsibility of states, cities, and Federal District to manage their own lists. Therefore, the selection of medicines at local level requires managers to define priorities and effectively allocate human and financial resources to strengthen and qualify the decentralization of the management^4^.

Brazil created its first LME over 50 years ago, and the Brazilian Ministry of Health invested on the development and publication of sequential Renames between 2002 and 2014, as well as on the reorientation of the qualification of pharmaceutical services^9^. Despite this, there are still many challenges regarding the selection of medicines in the Country^3^.

Marques and Zucchi^5^, for example, mention that few publications disclose the existence of PTC in outpatient and hospital services of SUS. In another national survey, entitled *Fala Essencial*, in which PS managers discuss their perception of the medicine selection process, of the 20 locations visited, only two states and five cities had LME^3^, in contradiction to the PNM^7^.

Thus, studies dealing with the selection of medicines in Brazil are scarce. The existing ones identified the lack of adoption of LME in the cities and in the Federal District, the inexpressive standardization of PTC, and the low rates of use of therapeutic forms and clinical protocols in primary health care^3^ ^,^ ^5^ ^,^ ^10^ ^,^ ^11^.

The *Pesquisa Nacional sobre Acesso, Utilização e Promoção do Uso Racional de Medicamentos – Serviços, 2015* (PNAUM – National Survey on Access, Use and Promotion of Rational Use of Medicines – Services, 2015) aimed to characterize the organization of pharmaceutical services in the primary health care of SUS, to promote the access and rational use of medicines, as well as to identify and discuss the factors that affect the consolidation of pharmaceutical services in the cities.

This article is part of PNAUM – Services and aimed to characterize the selection process in the primary health care of the Brazilian regions.

## METHODS

PNAUM – Services is a cross-sectional, exploratory, and evaluative study, consisting of an information gathering in a sample of cities and Federal District, representative to the five Brazilian regions. Several study populations were considered in the sampling, with samples stratified by the regions, which constituted the study domains. The PNAUM – Services methodology, as well as the sampling process, are described in detail by Álvares et al. (2017)^1^.

The data used in this study were collected from the databases of those responsible for pharmaceutical services, professionals responsible for the dispensing of medicines in *Unidades Básicas de Saúde* (UBS – Basic Health Units), and physicians from the UBS. The data were collected by interviews, adopting semi-structured scripts, via telephone or in person.

For determining the profile of respondents, we collected information on sex, age, education level, time in the position, and time of training. The characterization of the selection of medicines process was carried out by the analysis of the variables related to PTC, of the existence of standard operating procedures, List of Municipal Medicines (LMM), therapeutic form and/or clinical protocols, consensus or guidelines, and provision of non-standard medicines.

For this characterization, semi-structured questionnaires were adopted, which sought to describe the perceptions of respondents, i.e., the perception of coordinators of pharmaceutical services, of professionals responsible for the dispensation of medicines, and of physicians. The questions asked to these three actors were not coincident, but complementary, to show with greater comprehensiveness the selection of medicines scenario in the cities and Federal District.

Data analyses were performed using the SPSS 20.0 program, adopting the analysis module for complex samples. The variables were presented for Brazil and for each geographic region. The numerical variables were presented by the averages and by the 95% confidence interval (95%CI) of the averages. Categorical variables were presented by proportions and their 95%CI.

To evaluate the difference between ratios, we adopted the Chi-square test for complex samples. The assessment of the differences between averages was performed by generalized linear models, and the F-test with Bonferroni correction was adopted for multiple comparisons. The analyses considered significant had p ≤ 0.05.

PNAUM was approved by the National Research Ethics Committee, under Opinion no. 398,131, September 16, 2013, with clarification of the research’s objectives to the respondents and signing of the informed consent form.

## RESULTS

Among respondents, 1,558 were physicians and 1,139, professionals responsible for the dispensing of medicines in the pharmacies of the health units of the cities that composed the sample. We also conducted interviews by telephone with 506 coordinators of pharmaceutical services of the cities.

Table 1 presents the results of the profile of the professionals interviewed. Among those responsible for pharmaceutical services, the female sex prevailed (62%, 95%CI 56.9–66.9), with an average age of 34.8 years (95%CI 34.0–35.6), and higher education degree level (97.7%; 95%CI 95.8–98.7), among which 23.4% had attended a graduate course (95%CI 19.3–28.0). Almost all respondents (90.7%, 95%CI 87.3–93.4) declared to be pharmacists and with an average time in the position of over three years.

**Table 1:** Profile of participants of the selection process, interviewed by region in Brazil. National Survey on Access, Use and Promotion of Rational Use of Medicines – Services, 2015.

Profile of respondents	North	Northeast	Midwest	Southeast	South	Brazil	p
					
	% (95%CI)	% (95%CI)	% (95%CI)	% (95%CI)	% (95%CI)	% (95%CI)	
Professionals responsible for pharmaceutical services							
Sex (n = 506)							
Female	64.4 (54.0–73.6)	49.9 (39.0–60.8)	67.3 (57.3–75.9)	63.2 (53.1–72.3)	73.2 (63.7–81.0)	62.0 (56.9–66.9)	0.008
Age (n = 493)							
Average in years	34.2 (32.3–36.0)	37.1 (34.8–39.4)	31.8 (30.2–33.3)	35.5 (33.8–37.3)	35.6 (34.2–37.2)	34.8 (34.0–35.6)	0.003
Education level (n = 504)							
Higher education degree or more	94.4 (87.2–97.7)	97.5 (90.4–99.4)	92.7 (85.5–96.5)	99.0 (92.9–99.9)	99.0 (93.2–99.9)	97.7 (95.8–98.7)	0.122
Time in the position (n = 505)							
Average in months	26.3 (20.1–32.5)	43.0 (32.2–53.7)	31.4 (24.3–38.5)	54.2 (41.8–66.6)	49.5 (40.4–58.6)	40.9 (36.7–45.1)	< 0.001
Academic background (n = 505)							
Pharmacist	87.5 (78.8–93.0)	84.7 (75.0–91.1)	88.1 (80.0–93.2)	94.8 (88.0–97.8)	95.0 (88.6–97.9)	90.7 (87.3–93.4)	< 0.001
Professionals responsible for the dispensing of medicines							
Sex (n = 1,139)							
Female	77.5 (70.6–83.2)	80.9 (72.2–87.3)	68.8 (51.6–82.0)	69.7 (52.8–82.6)	87.4 (81.1–91.9)	77.4 (71.2–82.6)	0.087
Age (n = 1,137)							
Average in years	37.9 (36.5–39.3)	37.2 (34.8–39.6)	36.3 (33.6–39.0)	36.7 (36.2–41.2)	37.6 (34.9–40.3)	37.5 (36.5–38.6)	0.785
Education level (n = 1,139)							
Up to high school	68.3 (58.3–76.9)	72.7 (61.1–81.8)	27.0 (17.3–39.5)	28.3 (15.6–45.7)	42.2 (26.7–59.3)	50.3 (43.0–57.7)	< 0.001
Time in the position (n = 1,139)*							
Average in years	3.8 (3.4–4.3)	3.2 (2.5–3.9)	4.9 (3.4–6.3)	4.5 (3.6–5.4)	6.1 (4.4–7.9)	4.5 (4.0–5.0)	0.102
Academic background (n = 1,139)							
Pharmacist	8.9 (6.0–13.1)	2.6 (0.5–11.2)	32.7 (20.0–48.5)	33.4 (22.0–47.1)	13.8 (6.5–26.9)	16.6 (12.4–21.7)	< 0.001
Physicians							
Sex (n = 1,585)							
Male	53.0 (46.8–59.2)	57.4 (50.1–64.3)	54.2 (46.7–61.4)	57.6 (51.4–63.6)	51.5 (45.2–57.8)	55.8 (52.2–59.2)	0.509
Age (n = 1,531)							
Average in years	42.1 (40.6–43.6)	41.7 (39.5–43.9)	39.0 (37.0–41.0)	41.0 (39.4–42.6)	42.6 (40.8–44.4)	41.3 (40.5–42.1)	0.033
Time since graduation (n = 1,578)							
Average in years	13.8 (12.5–15.2)	13.2 (11.3–15.0)	11.2 (9.6–12.8)	14.6 (12.8–16.3)	14.9 (13.4–16.4)	13.5 (12.8–14.3)	0.005
Time in the position^*^ (n = 1,585)							
Average in months	27.9 (21.2–34.6)	31.8 (25.6–38.1)	37.8 (30.2–43.3)	48.5 (39.1–47.8)	51.5 (42.2–60.8)	39.3 (35.8–42.8)	< 0.001
Education level							
At least one residency	93.8 (89.9–96.2)	86.3 (80.4–90.6)	89.5 (83.8–93.3)	92.2 (88.2–95.0)	94.3 (90.5–96.6)	90.5 (88.0–92.5)	0.009

^*^ Average time in the position on the health unit where the interview was carried out. DNA: does not apply

Source: PNAUM – Services, 2015.

In the case of professionals responsible for the dispensing of medicines in the UBS, the female sex also prevailed (77.4%, 95%CI 71.2–82.6) and the average age was 37.5 years (95%CI 36.5–38.6). Concerning education level, 50.3% (95%CI 43.0–57.7) reported to have up to high school, and, of those who had higher education degree or graduate courses, only 16.6% (95%CI 12.4–21.7) were pharmacists. The average time in the position at the UBS was around four and a half years.

Regarding physicians, 55.8% were male (95%CI 52.2–59.2) and the average age was 41.3 years (95%CI 40.5–42.1). The time since graduation was asked only to physicians, whose average was 13.5 years (95%CI 12.8–14.3). The average working time in the position at the UBS where the interview was carried out was 39.3 months (95%CI 35.8–42.8), a little lower than that of the other interviewed professionals. 90.5% (95%CI 88.0–92.5) of physicians reported to have at least one residency course.

Few coordinators of pharmaceutical services reported the existence of a formally constituted PTC (12.5%; 95%CI 9.5–16.3). This proportion was lower in the Northeast and North regions (9.5% and 10.7%, respectively) and greater in the South (15.6%), although the difference is not statistically significant (Table 2).

**Table 2:** Characteristics of the medicine selection process, according to the perception of coordinators of pharmaceutical services, by region in Brazil. National Survey on Access, Use and Promotion of Rational Use of Medicines – Services, 2015.

Indicator of the selection of medicines	North	Northeast	Midwest	Southeast	South	Brazil	p
					
	% (95%CI)	% (95%CI)	% (95%CI)	% (95%CI)	% (95%CI)	% (95%CI)	
Existence of a formally constituted PTC (n = 503)	10.7 (6.0–18.3)	9.5 (4.8–18.0)	13.6 (8.2–21.9)	13.3 (7.8–21.7)	15.6 (9.8–23.9)	12.5 (9.5–16.3)	0.628
Existence of standard operating procedures (n = 491)	45.9 (35.6–56.6)	48.8 (37.7–60.0)	51.5 (41.4–61.4)	56.5 (46.5–66.1)	45.9 (36.4–55.7)	50.6 (45.3–55.8)	0.564
Existence of LME (n = 495)	70.6 (60.3–79.1)	90.8 (81.8–95.6)	82.9 (73.7–89.2)	86.9 (78.3–92.4)	82.0 (73.2–88.4)	85.3 (81.4–88.5)	0.077
Periodic update of the LME (n = 419)	82.9 (71.8–90.3)	83.8 (73.3–90.7)	83.1 (73.5–89.7)	81.2 (71.1–88.3)	73.1 (62.6–81.5)	80.4 (75.6–84.5)	0.347
Participation of the respondent in the development of the LME (n = 418)	90.5 (80.3–95.7)	87.0 (76.9–93.1)	84.7 (74.8–91.1)	90.1 (81.3–95.0)	88.0 (79.0–93.4)	88.2 (84.0–91.4)	0.592
Professionals request changes on the LME (n = 438)							0.804
Sometimes	33.8 (23.8–45.4)	30.2 (20.8–41.6)	31.3 (22.2–42.1)	29.2 (20.5–39.8)	33.6 (24.3–44.3)	31.0 (26.1–36.4)	
Rarely	23.5 (16.6–36.7)	25.8 (17.0–36.9)	25.8 (17.0–36.9)	24.1 (16.1–34.4)	23.1 (15.4–33.3)	24.1 (19.6–29.3)	
Never	33.5 (23.5–45.1)	31.4 (21.9–42.8)	31.4 (21.9–42.8)	35.9 (26.3–46.7)	23.8 (15.8–34.1)	31.1 (26.2–36.5)	
LME meets the health demands of the city (n = 426)							0.349
Completely	24.6 (15.6–36.5)	27.2 (18.1–38.7)	29.3 (20.4–40.1)	34.6 (25.1–45.5)	27.8 (19.2–38.2)	29.7 (24.7–35.1)	
Partially	75.4 (63.5–84.4)	72.8 (61.3–81.9)	70.7 (59.9–79.6)	65.4 (54.5–74.9)	71.1 (60.5–79.7)	70.1 (64.6–75.0)	
Provision of non-standard medicines (n = 506)	48.5 (38.3–58.7)	38.4 (28.4–49.6)	31.2 (22.7–41.0)	39.0 (29.8–49.2)	48.7 (39.1–58.4)	41.1 (36.2–46.3)	0.222
Existence of criteria for provision of non-selected medicines (n = 425)	64.8 (52.7–75.2)	56.1 (44.4–67.1)	72.8 (61.9–81.6)	73.9 (63.3–82.3)	71.1 (60.5–79.7)	67.0 (61.5–72.1)	0.081
Existence of a therapeutic form (n = 495)	21.0 (13.7–30.7)	27.9 (19.0–39.1)	16.2 (10.0–25.1)	23.8 (16.2–33.4)	18.3 (11.9–27.2)	22.9 (18.7–27.7)	0.252
Existence of clinical protocols (n = 487)	22.9 (15.3–32.9)	23.4 (15.2–34.3)	31.8 (23.3–41.8)	32.5 (23.7–42.8)	21.2 (14.2–30.4)	26.4 (22.0–31.3)	0.221

PTC: Pharmacy and Therapeutics Committee; LME: List of Essential Medicines of the cities and of the Federal District.

Source: PNAUM – Services, 2015.

Most coordinators (85.3%; 95%CI 81.4–88.5) stated that the city had an LME, of which 80.4% (95%CI 75.6–84.5) were considered to be periodically updated. Furthermore, approximately half of them declared to adopt standard operating procedures (SOP) to perform the selection of medicines in the cities (Table 2).

Still in Table 2, most coordinators (88.2%; 95%CI 84.0–91.4) claimed to have participated in the development of a LME in the city or Federal District. The request for changing the LME (inclusion and exclusion of medicines or intended use) seems to not be very frequent (sometimes 31.0%, rarely 24.1%, and never 31.1%), although the majority of coordinators assumed that the LME only partially (70.1%; 95%CI 64.6–75.0) meets the health demands of the city. Few coordinators reported the existence of therapeutic form or clinical protocols (22.9% and 26.4%, respectively). None of the results presented for these actors showed statistically significant differences between the regions of the Country.

According to the perception of the professionals responsible for the dispensing of medicines, most recognized the existence of a LME in the city (89.1%; 95%CI 83.8–92.8) and stated that the LME is present in the UBS for consultation by the health team (91.3%; 95%CI 86.5–94.5), especially in the Southeast region (96.5%) (Table 3).

**Table 3:** Characteristics of the medicine selection process, according to the perception of professionals responsible for the dispensing of medicines in the basic health unit, by region in Brazil. National Survey on Access, Use and Promotion of Rational Use of Medicines – Services, 2015.

Indicator of the selection of medicines	North	Northeast	Midwest	Southeast	South	Brazil	p
					
	% (95%CI)	% (95%CI)	% (95%CI)	% (95%CI)	% (95%CI)	% (95%CI)	
Existence of LME (n = 1,139)	84.6 (76.3–90.4)	88.7 (78.3–94.5)	92.0 (85.1–95.8)	86.6 (72.9–94.0)	95.7 (90.9–98.0)	89.1 (83.8–92.8)	0.189
The LME is available in the health units for consultation of professionals (n = 1,041)	80.2 (72.6–86.2)	88.6 (75.5–95.1)	90.1 (81.4–95.0)	96.5 (91.7–98.6)	93.1 (84.4–97.1)	91.3 (86.5–94.5)	0.057
The respondent knows the procedures for inclusion or exclusion of medicines in the LME (n = 930)	28.0 (21.3–35.9)	36.9 (27.9–46.9)	49.9 (34.1–65.7)	66.8 (50.9–79.7)	42.2 (27.8–58.1)	47.8 (40.6–55.2)	< 0.001
The respondent has already made a claim for inclusion/exclusion of medicines in the LME (n = 930)	31.0 (23.1–40.3)	42.1 (30.1–55.2)	66.2 (50.2–79.2)	45.7 (28.5–63.9)	44.9 (29.4–61.6)	44.4 (36.3–52.9)	0.528
The LME meets the health demands of the city (n = 930)	57.7 (49.1–65.9)	67.0 (55.1–77.0)	78.7 (64.1–88.4)	73.9 (55.1–86.7)	75.5 (60.5–86.1)	70.9 (63.4–77.3)	0.413

LME: List of Essential Medicines of the cities.

Source: PNAUM – Services, 2015.

The percentage of these professionals who knew the procedures for changing the LME was 47.8% (95%CI 40.6–55.2), with statistically significant difference between the regions (p < 0.001). According to Table 3, this proportion was lower in the North (28.0%; 95%CI 21.3–35.9) and higher in the Southeast (66.8%; 95%CI 50.9–79.7). The percentage of respondents who declared having already claimed for changes was 44.4% (95%CI 36.3–52.9), higher than the percentage of coordinators of pharmaceutical services. From the perspective of most of these professionals (70.9%; 95%CI 63.4–77.3), the LME meets the health demands of the city.

Physicians were asked about the characteristics of the selection of medicines, considering that they are the prescribers (Table 4). Only 27.2% (95%CI 23.7–30.9) of physicians reported to have knowledge of the procedures to change the LME, but 76.5% (95%CI 69.2–82.5) would have some claim to change it. Most reported to base their claims in clinical experiences (80.0%; 95%CI 71.7–86.4) or scientific articles (54.8%; 95%CI 45.5–63.7).

**Table 4:** Characteristics of the medicine selection process, according to the perception of physicians, by region in Brazil. National Survey on Access, Use and Promotion of Rational Use of Medicines – Services, 2015.

Indicator of the selection of medicines	North	Northeast	Midwest	Southeast	South	Brazil	p
					
	% (95%CI)	% (95%CI)	% (95%CI)	% (95%CI)	% (95%CI)	% (95%CI)	
Knowledge of the respondent regarding the procedures for inclusion or exclusion of medicines in the LME (n = 1.254)	25.8 (19.6–33.2)	27.7 (20.8–35.7)	21.2 (23.8–36.6)	29.8 (23.8–36.6)	24.2 (18.8–30.6)	27.2 (23.7–30.9)	0.542
Existence of a claim for inclusion/exclusion of medicines in the LME (n = 282)	80.4 (64.3–90.3)	80.3 (77.9–92.1)	89.5 (75.8–95.9)	67.9 (54.0–79.2)	75.6 (62.3–85.3)	76.5 (69.2–82.5)	0.167
Base for solicitation^a^							
Scientific article (n = 204)	40.4 (25.1–57.7)	44.0 (27.9–61.5)	52.9 (33.3–71.6)	66.9 (50.0–80.4)	63.2 (46.2–77.5)	54.8 (45.5–63.7)	0.095
Scientific events (n = 203)	34.4 (20.3–51.9)	16.2 (7.1–32.8)	35.6 (19.3–55.9)	35.8 (21.6–53.1)	40.8 (25.5–58.1)	29.0 (21.6–37.7)	0.060
Journals (n = 203)	17.9 (8.3–34.6)	14.4 (5.8–31.6)	36.5 (20.1–56.7)	33.2 (20.3–49.4)	22.8 (12.0–39.0)	23.1 (16.6–31.2)	0.145
Media (television, magazines, radio, internet etc.) (n = 202)	11.8 (4.3–28.5)	6.8 (1.7–23.6)	6.0 (1.5–20.7)	8.4 (2.5–24.6)	6.9 (2.1–20.3)	7.6 (3.9–14.4)	0.933
Visit of representative/propagandist (n = 201)	11.8 (4.3–28.5)	14.0 (5.5–31.4)	10.9 (3.4–29.5)	9.2 (3.2–23.7)	25.7 (13.2–44.0)	14.4 (9.0–22.1)	0.261
Clinical experience (n = 200)	85.4 (70.1–93.6)	82.2 (64.5–92.1)	83.2 (62.6–93.6)	80.7 (64.2–90.6)	71.3 (54.3–83.8)	80.0 (71.7–86.4)	0.546
Existence of LME (n = 1,451)	90.9 (86.1–94.2)	94.8 (90.5–97.3)	92.8 (87.4–96.0)	95.5 (92.0–97.5)	98.1 (95.1–99.3)	95.3 (93.5–96.6)	0.092
Participation in the development of the LME (n = 1,311)	10.9 (7.1–16.5)	13.7 (8.9–20.4)	12.8 (8.6–18.7)	17.4 (12.5–23.8)	16.1 (11.8–21.5)	15.1 (12.5–18.3)	0.523
The LME meets completely the health demands of the city (n = 1,296)	14.2 (9.7–20.2)	12.5 (7.9–19.1)	10.0 (5.9–16.6)	15.2 (10.7–21.2)	10.7 (7.5–15.1)	13.0 (10.5–15.9)	0.223
Knowledge of the existence of the LME (n = 1,381)	93.9 (89.7–96.4)	95.3 (90.7–97.6)	95.3 (90.7–97.7)	95.9 (92.4–97.8)	97.7 (94.5–99.1)	95.9 (94.1–97.1)	0.502
Form of access to the LME: ^b^							
Available in the medical office (n = 1,303)	72.1 (64.9–78.3)	74.6 (67.3–80.8)	74.3 (66.8–80.6)	81.3 (75.6–85.9)	79.6 (73.8–84.4)	77.6 (74.3–80,6)	0.181
Available on the internet (n = 1,172)	17.7 (12.6–24.3)	11.0 (6.8–17.2)	21.3 (14.9–29.4)	24.7 (19.3–31.0)	22.8 (17.4–29.3)	18.6 (15.9–21.7)	0.001
Available at the pharmacy (n = 1,259)	66.8 (59.4–73.4)	63.3 (55.0–70.9)	74.0 (65.8–0.7)	66.3 (59.0–73.0)	62.9 (56.0–69.4)	65.0 (61.0–68.8)	0.551
Available by the SMS or SES-DF (n = 1,149)	49.2 (41.7–56.7)	51.5 (42.9–60.0)	51.4 (42.5–60.3)	49.8 (41.9–57.7)	53.6 (46.7–60.5)	51.3 (47.1–55.0)	0.898
Availability of TF, Consensus, Guidelines, or Lines of Care in the medical office	51.6 (45.0–58.2)	46.7 (39.3–54.3)	41.8 (34.6–49.3)	54.7 (48.0–61.3)	50.1 (43.6–56.6)	49.9 (46.3–53.6)	0.191
The patient requests a change of medicine:							
Always, repeatedly, or sometimes (n = 1,578)	56.8 (50.3–63.0)	63.3 (55.9–70.0)	67.5 (60.7–73.8)	71.4 (65.3–76.8)	68.2 (61.5–74.2)	66.5 (63.0–69.8)	0.065

LME: List of Essential Medicines of the cities; SMS: Municipal Secretariat of Health; SES-DF: State Secretariat of Health of the Federal District; TF: Therapeutic Form.

^a^ Type of material consulted to make the request for inclusion in the standardized List of Medicines: scientific article, events, journals, media (magazines, television, radio, and internet); visit of representative/propagandist, clinical experience.

^b^ Options of access to the standardized List of Medicines mentioned by physicians: in the medical office, on the internet, at the pharmacy, at the Municipal Secretariat of Health.

Source: PNAUM – Services, 2015.

Most physicians declared to know of the existence of the LME (95.3%; 95%CI 93.5–96.6), without significant differences between the regions of the Country (p = 0.092) (Table 4), with lower proportions in the North and Midwest regions (90.9% and 92.8%, respectively) and higher in the South region (98.1%). The form of access to the lists varied significantly: availability in the medical office (77.6%; 95%CI 74.3–80.6); in the UBS pharmacy (65.0%; 95%CI 61.0–68.8); or in the secretariat of health (51.3%; 95%CI 47.1–55.0); and lower internet access (18.6%; 95%CI 15.9–21.7).

The availability of therapeutic form or consensus, clinical practice guidelines or lines of care in the medical offices of the UBS was mentioned by half of physicians (49.9%; 95%CI 43.6–53.6) (Table 4).

An interesting fact was that the physicians declared that many patients request a change of medicines always, repeatedly, or sometimes (66.5%; 95%CI 63.0–69.8). Few physicians considered that the LME meets the health demands of the city (13.0%; 95%CI 10.5–15.9). Unlike the coordinators of pharmaceutical services, few physicians of the UBS declared to participate in the development of the LME (15.1%; 95%CI 12.5–18.3).

## DISCUSSION

The data collected by PNAUM – Services are unseen to the Country regarding the characteristics of the medicine selection process in Brazilian cities and in the Federal District. PNAUM is a pioneer descriptive research with regional and national representativity and approach based on the perspectives of different professionals.

We noticed that the average age of all the interviewed professionals was 38 years, higher for physicians (as expected due to the time of undergraduate courses and residency of the professional) and lower for coordinators of pharmaceutical services. This reflects that the sample represents adults in a phase of professional improvement and increase of maturity.

Working time in the position was low and quite similar between physicians and coordinators of pharmaceutical services, which could represent a certain similarity with the political-electoral cycle experienced by the Brazilian cities. The low average age of professionals responsible for dispensation could be related to other factors, such as the education level required for this occupation.

The low education level among the professionals responsible for the dispensing of medicines (50.3% up to high school) is a point that deserves reflection, as it may represent a professional who is not adequately prepared to provide guidance on the correct use of medicines. This shows the weakness that the patient of SUS primary health care has to achieve safe and responsible use of medicines, as referred to in PNM^7^ (in its guidelines, priorities and responsibilities of each federated entity) and in the strategic axis XIII of the *Política Nacional de Assistência Farmacêutica* (PNAF – National Policy of Pharmaceutical Services)^8^.

The selection process for medicines in the context of the logistics cycle of pharmaceutical services is a fundamental and little studied stage that can influence the promotion of a safer, more effective, and less costly use of medicines^4^ ^,^ ^10^.

We highlight that the PNM^7^ establishes that the management of pharmaceutical services must be decentralized, with the selection, programming, and procurement of medicines made based on epidemiological criteria to better meet local needs for medicines. For the Brazilian cities and Federal District, therefore, the effective and perennial selection of medicines is an important factor in the management of financial resources and in the access to essential medicines for the patients of health services.

Despite this premise, Brazilian states, cities, and Federal District have experienced difficulties in conducting the selection process, considering the relatively recent context of full decentralization of pharmaceutical services^3^. Therefore, the structuring of a multidisciplinary PTC, including health professionals at various levels of health care and management, is scarce in the Country^2^ ^,^ ^4^.

The results of this research show two concerning aspects regarding compliance to pharmaceutical policies^a,b^. Only 12.5% of respondents described the existence of a formally constituted PTC, and only 27.2% of physicians reported to know the criteria for changing the LME, such as inclusion or exclusion of medicines.

However, the existence of a LME is quite recognized by the interviewed professionals (85.3% among those in charge of pharmaceutical services, 89.1% among those responsible for the dispensing of medicines, and 95.3% among physicians), which denotes that there is no technical process free from conflict of interest in the selection of medicines. If there is no constituted PTC, how is the process of selection of medicines conducted? The non-existence of a formalized PTC opens space for the insertion of medicines in the LME to be made without criteria of quality, safety, and effectiveness, as established by the World Health Organization^2^ ^,^ ^12^.

The ability of the LME to meet local needs for medicines can be considered quite low in the perspective of 13% of primary health care physicians, 29.7% of coordinators of pharmaceutical services, and 70.9% of professionals responsible for the dispensing of medicines.

This discrepancy in the results can show different views between these professionals. Physicians may be more susceptible to the propagandists of pharmaceutical companies to prescribe medicines that are not listed in the LME, as this advertising is not always accompanied by the criteria of essentiality that must guide the selection of medicines^11^. At the same time, these prescribers can adapt their prescription to the availability of medicines at the UBS, which can thus influence the perception of professionals responsible for the dispensing of medicines on the UBS. In addition, when prescribing a medicine that is not listed in the LME, the physician should guide patients to purchase the medicines and, therefore, this demand would not be perceived by those dispensing the medicines. Anyway, the results found for physicians and professionals who in charge of the dispensing of medicines in the UBS are antagonistic and deserve to be investigated with more depth.

Still concerning the prescribers, among those who knew the procedures for changing the LME, 76.5% had already claimed for the inclusion or exclusion of medicines, which may be natural, as they are actors who are closely related to the therapeutic choice. However, little more than half of them (54.8%) reported doing this request based on scientific articles. Considering that the selection of medicines should be based on the best evidence, this can be considered worrisome^2^ ^,^ ^4^.

The small availability of therapeutic forms and consensus, guidelines and/or clinical protocols also stands out – estimated, respectively, at 22.9% and 26.4%, from the perspective of the coordinators of pharmaceutical services, and at 49.9% in the perspective of primary health care physicians. According to Wannmacher^11^, a LME must be complemented by a therapeutic form and clinical protocols, to influence the rational selection and guidance for the use of medicines adopted in the prevention and treatment of diseases that are prevalent and relevant to the Country.

The cross-sectional design is a limitation of the study, which, although being able to show the reality of the medicine selection process in the Country, is not specific regarding the evolution of this process. Another limitation refers to the significant percentages of non-response to some variables in the sample. However, the originality of the research, along with the size and scope of the sampling, allow us to meet the perspectives of different health professionals and provide evidence that can be used for the improvement of the actions from the guidelines and strategic goals of PNM^7^ and PNAF^8^, respectively. Improving the process of selection of medicines in the cities, allocating apt professionals to the dispensing of medicines, and facilitating the access by primary health care professionals to the guidelines and evidence-based clinical protocols will provide a better use of public resources and the safe and responsible use of medicines in SUS.

## References

[1] Álvares J, Alves MCGP, Escuder MML, Almeida AM, Izidoro JB, Guerra AA (2017). Pesquisa Nacional sobre Acesso, Utilização e Promoção do Uso Racional de Medicamentos: métodos. Rev Saude Publica.

[2] Holloway K, Green T (2003). Drug and therapeutics committees: a practical guide.

[3] Magarinos-Torres R, Pepe VLE, Oliveira MA, Osorio-de-Castro CGS (2014). Medicamentos essenciais e processo de seleção em práticas de gestão da Assistência Farmacêutica em estados e municípios brasileiros. Cienc Saude Coletiva.

[4] Marin N, Luiza VL, Osorio-de-Castro CGS, Machado-dos-Santos S (2003). Assistência farmacêutica para gerentes municipais.

[5] Marques DC, Zucchi P (2006). Comissões farmacoterapêuticas no Brasil: aquém das diretrizes internacionais. Rev Panam Salud Publica.

[6] Messeder AM, Osorio-de-Castro CGS, Luiza VL (2005). Mandados judiciais como ferramenta para garantia do acesso a medicamentos no setor público: a experiência do Estado do Rio de Janeiro, Brasil. Cad Saude Publica.

[7] Ministério da Saúde (BR), Secretaria de Políticas de Saúde, Departamento de Formulação de Políticas de Saúde (2001). Política Nacional de Medicamentos.

[8] Ministério da Saúde (BR), Conselho Nacional de Saúde (2004). Resolução nº 338 de 06 de maio de 2004. Aprova a Política Nacional de Assistência Farmacêutica. Diario Oficial Uniao.

[9] Portela AS, Leal AAF, Werner RPB, Simões MOS, Medeiros ACD (2010). Políticas públicas de medicamentos: trajetória e desafios. Rev Cienc Farm Basica Apl.

[10] Santana RS, Jesus EMS, Santos DG, Lyra DP, Leite SN, Silva WB (2014). Indicadores da seleção de medicamentos em sistemas de saúde: uma revisão integrativa. Rev Panam Salud Publica.

[11] Wannmacher L (2010). Seleção de medicamentos essenciais: propósitos e consequências. Rev Tempus Actas Saude Coletiva.

[12] World Health Organization (2013). WHO model list of essential medicines:18th list (April 2013).

